# A simple Gateway-assisted construction system of TALEN genes for plant genome editing

**DOI:** 10.1038/srep30234

**Published:** 2016-07-25

**Authors:** Hiroaki Kusano, Hitomi Onodera, Miho Kihira, Hiromi Aoki, Hikaru Matsuzaki, Hiroaki Shimada

**Affiliations:** 1 Department of Biological Science and Technology, Tokyo University of Science, Katsushika, Tokyo 125-8585, Japan

## Abstract

TALEN is an artificial nuclease being applied for sequence-specific genome editing. For the plant genome editing, a pair of TALEN genes is expressed in the cells, and a binary plasmid for *Agrobacterium*-mediated transformation should be assembled. We developed a novel procedure using the Gateway-assisted plasmids, named Emerald–Gateway TALEN system. We constructed entry vectors, pPlat plasmids, for construction of a desired TALEN gene using Platinum Gate TALEN kit. We also created destination plasmid, pDual35SGw1301, which allowed two TALEN genes to both DNA strands to recruit using Gateway technology. Resultant TALEN genes were evaluated by the single-strand annealing (SSA) assay in *E. coli* cells. By this assay, the TALENs recognized the corresponding targets in the divided luciferase gene, and induced a specific recombination to generate an active luciferase gene. Using the TALEN genes constructed, we created a transformant potato cells in which a site-specific mutation occurred at the target site of the *GBSS* gene. This suggested that our system worked effectively and was applicable as a convenient tool for the plant genome editing.

Targeted genome modifications will become routine in near future in crop plants^1^. A number of new genome editing technologies have been developed, including zinc finger nucleases (ZFNs), transcription activator like effector nucleases (TALENs), and clustered regulatory interspaced palindromic repeats/Cas9 (CRISPR/Cas9) systems^2^. These sequence-specific nucleases create double strand breaks (DSBs) at the targeted loci^3^. They induce subsequent repairs of the genomes in living cells, such as non-homologous end joining (NHEJ) and homologous recombination (HR). These events cause site-directed mutagenesis and/or integrations of desired DNA fragments at the targeted sites^4^.

TALEN is a sequence-specific nuclease created by fusing transcription activator like effectors (TALEs) to the catalytic domain of the *Fok*I endonuclease^5^. TALEN shows high genome editing activity with low toxicity in human cells^6^, and leads to efficient plant genome engineering^7^. The DNA binding domains of TALENs contain highly homologous 34 amino acid direct repeats (34-aa repeats) determining specificity^8^. In the DNA binding domain, there are specific binding motifs, two hypervariable amino-acid residues known as repeat-variable di-residues (RVDs), each of which recognizes one base pair in the target DNA^9^. Among the RVDs, NI, HD, NH/NK and NG strictly recognize adenine, cytosine, guanine, and thymine nucleotides, respectively, whereas some RVDs, such as NN and NS, show degenerated specificity to guanine and adenine nucleotides^8^. If high specificity for guanine is required, the RVDs NH or NK are preferred, but RVD NN should be chosen if weak overall efficiency of the TALE needs to be increased^10^. To construct the DNA binding motifs of TALEN for the desired target sequence, the efficient systems, such as Platinum Gate system, have been developed^11^. Gateway-assisted assemble systems have been applied for the animal genome editing. Using this system, a single plasmid containing two TALEN genes that were driven by the CAG promoter and human EF1α promoter, respectively, were developed for editing of the vertebrate genome^12^.

In case of plant genome editing, a pair of TALEN genes needs to be introduced and expressed in the cells. However, it requires bothersome works to assemble them in a binary vector for the *Agrobacterium*-mediated transformation of the plant cells. Here, we propose a Gateway-assisted construction of TALEN genes, which leads to rapid creation of a desired binary plasmid.

## Results

### Development of Emerald-Gateway TALEN system

**Figure 1:**
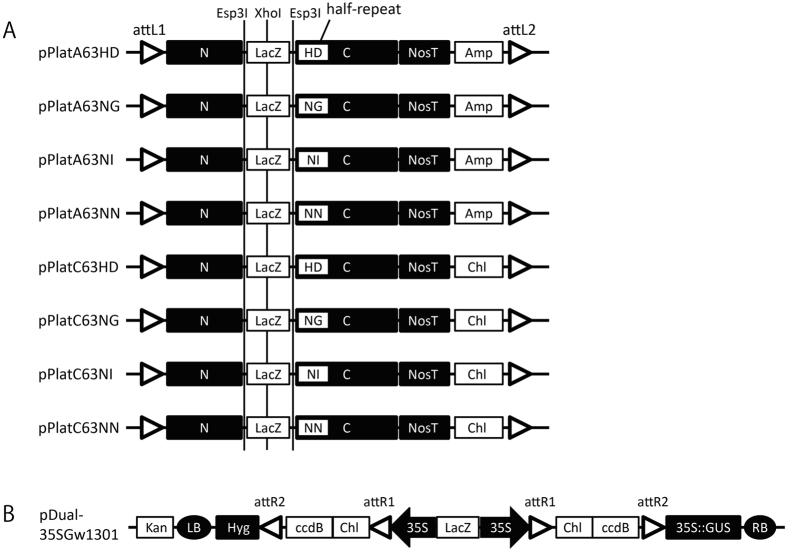
Structure of the plasmids for Emerald TALEN Gateway system. (**A**) Structure of a series of the entry vectors, pPlat plasmids. They contain different C-terminal RVD regions composed of the half repeat of the DNA recognition repeats, HD, NG, NI, and NN, beside the cloning sites, which was specific to the cytosine, thymine, adenine, and guanine, respectively. 34-aa repeat region of recognition domain in the TALENs is introduced using Platinum Gate TALEN kit, and replaced by the lacZ gene lying between two *Esp*3I sites. Filled boxes “N” and “C” indicate the 5′ and 3′ portions of “the 136/63 Scaffold” of Platinum TALEN ORFs^11^. attL1 and attL2: recombination sites in the entry vectors for Gateway LR reaction; Amp and Chl: ampicillin and chloramphenicol resistant genes; LacZ: *E. coli* LacZ–α gene; NosT: NOS-terminator. (**B**) Structure of the destination vector, pDual35SGw1301. A pair of TALEN genes is introduced between attR1 and attR2. The generated TALEN genes were driven by 35S promoter. LB and RB: left and right borders for the T-DNA; 35S::GUS, GUS gene driven by 35S promoter; Kan, Chl, Hyg, and ccdB: kanamycin resistant, chloramphenicol resistant, hygromycin resistant, and ccdB genes, respectively; attR1 and attR2: acceptor sites for Gateway LR reaction.

**Figure 2:**
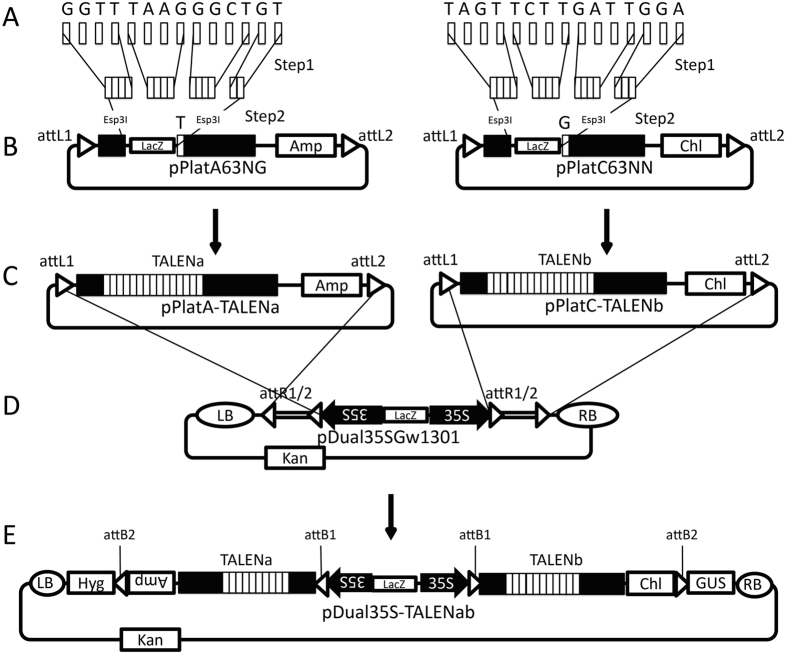
Scheme of construction of the binary plasmid containing the TALEN genes for the potato *GBSS* gene. The modules of the 34-aa repeats corresponding to the target sequences (panel A) were connected, and then introduced into pPlatA63NG and pPlatC63NN, member of pPlat plasmids (panel B) using Platinum Gate TALEN kit, to construct entry plasmids. Resultant pPlatA-TALENa and pPlatC-TALENb (panel C) were subjected to react with pDual35SGw1301, a destination plasmid (panel D), by LR clonase. The resultant binary plasmid, pDual35S-TALENab (panel E) contained recombinant TALEN genes. A, T, G, and C, indicate the modules corresponding to the 34-aa repeat, each of which recognizes Adenine, Thymine, Guanine and Cytosine, respectively. TALENa and TALENb show the resultant TALEN genes. Structure of pPlat plasmids, pDual35SGw1301, and related plasmids refers to Fig. 1.

We employed the Platinum Gate TALEN kit^11^ to adapt for the Gateway technology that enabled rapidly assembling the desired TALEN genes in a binary vector. We produced a series of the pPlat vectors, which functioned as Gateway entry vectors containing a cloning site between two *Esp*3I sites at the region corresponding to the DNA-binding domain in the platinum TALEN (Fig. 1A). For the destination vector, we created a plasmid, pDual35SGw1301, which contained two cloning cassettes composed by the cauliflower mosaic virus (CaMV) 35S promoter followed by the recombination sites for the Gateway technology (Fig. 1B). Pieces in the DNA-binding domain corresponding to the desired target nucleotide sequence were inserted into an appropriate pPlat vector according to the procedure of the Platinum Gate TALEN kit. A pair of the resultant TALEN genes was simultaneously transferred into the destination vectors by the Gateway system. In this system, it is expected that these genes in the resultant plasmid are expressed under 35S promoters (Fig. 2). We named our system using pPlat plasmids and the destination vector as “Emerald-Gateway TALEN system”.

### Construction of the TALEN genes

**Figure 3:**
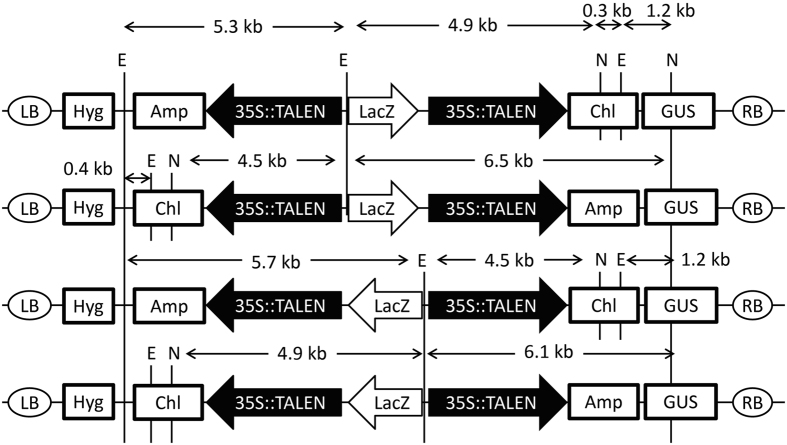
Structure of the binary plasmid containing TALEN genes for the *GBSS* genes. Arrow boxes show direction of transcription of the genes. 35S::TALEN indicates each of TALEN genes following 35S promoters. Different types of T-DNAs were generated by the Gateway reactions according to the positions of recombination. LB and RB: left and right borders for the T-DNA. Hyg, Amp and Chl: hygromycin, ampicillin, and chloramphenicol resistant genes, respectively; LacZ: *E. coli* LacZ–α gene; GUS: GUS gene; N and E: recognition sites of *Nco*I and *Eco*RI. Sizes of the fragments are indicated.

There are eight kinds of pPlat plasmids that contain different C-terminal RVDs at the 34-aa repeat in the divided TALEN gene and each of two resistant markers (Fig. 1A). We designed two sets of target sequences of TALENs, which were located in the potato granule-bound starch synthase (*GBSS*) gene. We obtained the desired TALENs in a pPlat plasmid using the Platinum Gate system (see Methods). The resultant plasmids for a pair of the TALEN genes were subjected to the Gateway-mediated assembly in pDual35SGw1301. Our system generated four types of destination plasmids, which showed differences in direction of the TALEN genes and attached resistant genes (Fig. 3). The desired plasmid including a heterologous pair of TALEN genes was selected by resistance to Kanamycin, Chloramphenicol, and Ampicillin, and by formation of blue colonies on the X-Gal plates.

### Evaluation of the constructed TALENs

We performed single-strand annealing (SSA) assay, which was widely used for determination of double-strand DNA breakage^13^, coupled with a luciferase (Luc) assay. We evaluated the function of the constructed TALENs by SSA assays using the target sequences in *GBSS* gene, which were localized in the different points of the gene (see Supplemental Fig. 1). The target sequences were put into the divided Luc gene, and they were introduced into *E. coli* cells together with the TALEN genes. After the target sequence was digested by the appropriate TALENs, it was expected that the mature Luc gene was reconstructed from the divided Luc gene by the simultaneous recombination, and that the Luc activity arose in the cells.

**Figure 4:**
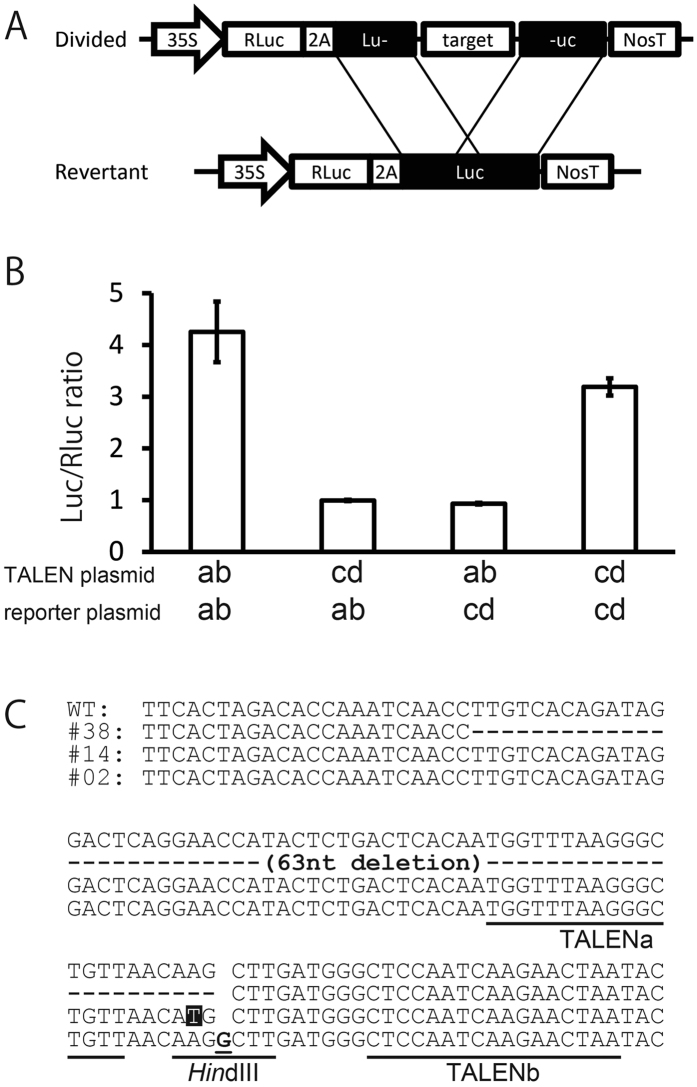
Results of SSA assay in *E. coli* cells and detection of mutation in the potato callus. (**A**) Structure of the plasmids for SSA assay containing a divided Luc (firefly luciferase) gene and the expected gene that will be generated by the specific recombination on the overlapped regions. “Divided” indicates the structure of the reporter plasmid that contains a fusion gene for RLuc and 2A peptide followed by the divided Luc. The divided Luc gene is inactivated by insertion of the target sequence of TALENs (shown by “target”). Lu- and -uc show parts of divided Luc genes, in which 430-nucleotides region are repeated. “Revertant” indicates the reporter gene, which will be generated after the appropriate TALEN reaction will induce the site-specific gene recombination at the overlapped region. 35S: 35S promoter; RLuc, *Renilla* luciferase; 2A, self-cleavable 2A peptide; target, the target sequence of TALENs; NosT, NOS terminator. (**B**) Results of SSA assays. Assays were performed using a set of plasmids, a TALEN plasmid and a reporter plasmid, pSSARLab or pSSARLcd, which are corresponding to “Divided” on the panel A. pSSARLab contains the target sequence by TALENa and TALENb that are encoded in TALENab as a TALEN plasmid. pSSARLcd contains those of TALENc and TALENd in TALENcd. “ab” and “cd” show the names of TALEN plasmids and reporter plasmids used. Dual luciferase assays were performed using the cell extract of *E. coli* transformed both by the plasmids for the SSA assay and TALENs. Relative values of the Luc activity to the RLuc activity are shown (n = 5). (**C**) DNA sequences of the regions corresponding to the target site of the TALENs. WT, #38, #14, and #02 show the nucleotide sequences of the wild-type gene and three mutants of the potato callus, respectively. Gaps indicate the region of nucleotide deletion and a site of insertion. An underlined letter shows the inserted nucleotide in #02, and a reversed letter indicates an A to T substitution. Bars show target sites of TALENs, TALENa and TALENb, and *Hin*dIII site.

The significantly high level of Luc activity was detected in *E. coli* cells harboring the divided Luc gene together with the appropriate TALEN genes (Fig. 4B, combination of TALEN “ab” and a reporter plasmid “ab”, and that of TALEN “cd” and a reporter “cd”), whereas a basal level of Luc activity was shown in the transformant cells containing the divided Luc gene with the sequence that was not the target of the introduced TALENs (Fig. 4B, combination of TALEN “cd” and a reporter “ab”, and that of TALEN “ab” and a reporter “cd”). These results indicate that the TALENs specifically recognized the target sequence, and induced an SSA event. This suggested that these TALENs were efficiently functioned.

Using the TALEN genes constructed, we transformed potato cells to induce a mutation at the target site of the *GBSS* gene. From the transgenic callus cells, we extracted the genomic DNA, and amplified the fragment corresponding to the *GBSS* gene in which the target sequence was included. We detected the fragments lacking the *Hin*dIII digestion site at the target site of the TALENs. We found three kinds of genome modifications that had occurred independently. We detected 63 nucleotides deletion, one nucleotide insertion, and one nucleotide substitution at the *Hin*dIII site (Fig. 4C). This suggests that the site-specific mutation occurred at the target site in the potato cells *in planta*.

## Discussion

We developed a novel TALEN construction system, named Emerald Gateway TALEN system, which was customized for Gateway system using Platinum Gate TALEN kit. Our system composed of two-step procedures to produce a pair of TALEN genes for the plant genome editing. In the first step, a pair of entry plasmids containing each of the desired TALEN genes was constructed using pPlat plasmids. The resultant TALEN genes were then introduced into a binary vector as a destination plasmid by the second step to generate a desired binary plasmid (Fig. 2).

A TALEN gene includes highly repetitive sequences, and therefore it is difficult to construct a plasmid consisting of the desired structure. Our system needs a destination vector that contains two sets of expression units composed of appropriate promoters and following acceptor sequences of Gateway attR1/2, to which each of desired TALEN genes is inserted. pPlat plasmids are entry vectors that are available for construction of a binary plasmid by Gateway technology (Fig. 2). Once the TALEN genes were constructed using pPlat plasmids, they can be efficiently transferred into a destination vector.

In this study, we prepared the desired binary plasmids in a week, suggesting that our system allows high efficiency and reliability for construction of the binary plasmid that contains the desired TALEN genes without requirement of much purification processes on the intermediate products. A desired pair of TALEN genes was easily obtained by a quadruple selection by resistance to three antibiotics, and by blue-white selection using X-Gal, to obtain. We propose a general protocol for construction of the TALENs by our system (see Supplemental materials). This procedure results in reduction of labors compared with those required in the conventional TALEN gene construction processes, and leads to timesaving experiment on the plant genome editing.

SSA assay using *E. coli* cells detected the Luc activity that was induced by the mutagenesis at the specific target sequence (Fig. 4). It was known that the 35S promoter worked in *E. coli* cells^14^. This result suggests that the resultant TALEN genes effectively functioned for the genome editing events in the cells. In addition, we obtained the transformant potato cells using the constructed TALEN genes (Fig. 4C). These facts show that our system is reliable for the tools to construct the active TALEN genes for the plant genome editing.

In this work, we employed CaMV 35S promoter for expression of TALEN genes in the destination vector. These CaMV 35S promoter regions were located between *Xba*I and *Kpn*I sites in the destination plasmid (Fig. 2E), and easily replaced by any other promoters. This suggests that an appropriate destination vector can be constructed conveniently depending on the demands.

## Methods

### Materials

We used *E. coli* strain NEB stable (New England Biolab, MA, USA) that is expected to reduce unexpected recombination in repeat sequences for the plasmid construction. Platinum Gate TALEN kit was obtained from Addgene (#1000000043, Cambridge, MA, USA).

### Construction of vector plasmids

Entry plasmids, named as a series of pPlatA and pPlatC, were constructed using pDONR207 (Invitrogen, Carlsbad, USA). Depending to the resistant genes for the selection marker, we generated two different types of plasmids. Desired TALEN genes were employed in the entry plasmids using Platinum Gate TALEN kit^11^. A destination plasmid, pDual35SGw1301, was constructed using pGWB2^15^ and pCAMBIA1301 (CAMBIA, Australia). Detailed methods of the plasmid construction were described in Supplemental Methods.

### Construction of TALEN genes

In this work, following sequences contained in the potato *GBSS* gene (acc. no. NW_006238976) was used as the targets of TALENs: 5′-TGGTTTAAGGGCTGTTaacaagcttgatgggCTCCAATCAAGAACTAA, and 5′-TGTGATCCGCTACTTTAtctgcaggtcaaagtTGGAGACAGCATTGAAA (capital letters indicate the sequence recognized by the corresponding TALENs), respectively. The regions of the 34-aa repeats containing appropriate RVDs for the recognition sites of the TALENs were constructed using the Platinum Gate TALEN kit^11^ with following modifications. We used pPlat plasmids as the acceptor plasmids. The resultant TALEN genes were assembled into pDual35SGw1301 by Gateway LR clonaseII (Invitrogen). The resultant plasmids were prepared using a Fastgene miniprep kit (Nippon Genetics, Tokyo, Japan) from the *E. coli* cells that were selected as blue colonies on the plate containing X-Gal with resistance to Kanamycin, Ampicillin and Chloramphenicol.

### Construction of plasmids for SSA assays

The SSA vector plasmid, pSSARL-GwBP, was constructed using pBluescriptII SK+ by introduction of CaMV 35S promoter followed by the coding sequences for the *Renilla* luciferase, self-cleavable 2A peptide^16^, the divided Luc, and Nos terminator. The regions for 35S promoter and RLuc were amplified by PCR from pCAMBIA1301 and pRL-null (Picagene Dual Seapansy Luminescence kit, Toyo-B-net, Tokyo, Japan), respectively. The fragment of 2A and the divided Luc gene was chemically synthesized. This gene contained the coding region for the first from two-thirds of the Luc gene (from 1 to 1035 nucleotide position) and the region behind the third of the Luc gene (from 605 and later), in which the 430 nucleotides was repeated in the border region. Inside the divided Luc, there was a gateway attP site to which the desired sequence was introduced by BP clonase to generate pSSARL-GwBP. The target fragment (50 nucleotides) of the TALENs for the potato *GBSS* gene was chemically synthesized, and introduced into pSSARL-GwBP by the reaction of BP clonase, to generate each of the SSA target plasmids, pSSARLab and pSSARLcd. Detailed methods of the plasmid construction were described in Supplemental Methods.

### SSA assay

*E. coli* cells were transformed by introduction of the plasmids each of which contained for the SSA target sequence and the TALEN genes. The transformant cells showing resistance both to Kanamycin and Ampicillin were grown for 16 hours in a medium containing 1% peptone, 0.5% yeast extract, and 0.5% NaCl at 37 °C. The Luc activity was determined by the fluorometric assay method using Picagene Dual Seapangy Luminescence kit (Toyo-B-net, Japan) and Luminometer ARVO light (PerkinElmer, MA). *Renilla* luciferase activity was measured as the internal control. Luc activities derived from the introduced SSA target plasmid were normalized as the relative values to the *Renilla* luciferase activity.

### Detection of site-specific mutation in the target gene

A fragment corresponding to the region including the target site of the TALEN genes was amplified by PCR using a high-fidelity KOD FX neo DNA polymerase (Toyobo) from the genomic DNA of the transformant potato callus, which was created by the *Agrobacterium*-mediated transformation method using the appropriate TALEN genes. The amplified fragment was cleaved by *Hin*dIII to enrich the population of fragments lacking the *Hin*dIII site. The resultant fragments were introduced into pBluescriptII SK + (Toyobo), and cloned in *E. coli* cells. From the generated *E. coli* cells, the fragment containing the target site was amplified by PCR. Site-specific mutation at the target site of the TALENs was determined by generation of the fragment that was not digested by *Hin*dIII. Detailed methods of the plasmid construction were described in Supplemental Methods.

## Additional Information

**Accession Codes**: Accession numbers of the nucleotide sequences of the plasmids produced in this work are LC122497 (pPlatA63HD), LC122498 (pPlatA63NG), LC122499 (pPlatA63NI), LC122500 (pPlatA63NN), LC122501 (pPlatC63HD), LC122502 (pPlatC63NG), LC122503 (pPlatC63NI), LC122504 (pPlatC63NN), LC122496 (pDual35SGw1301), and LC122270 (pSSARL-GwBP), respectively.

**How to cite this article**: Kusano, H. *et al*. A simple Gateway-assisted construction system of TALEN genes for plant genome editing. *Sci. Rep.* **6**, 30234; doi: 10.1038/srep30234 (2016).

## Supplementary Material

Supplementary Information
