# Supplementary material for: A simple Gateway-assisted construction system of TALEN genes for plant genome editing
Source: Sci Rep. 2016 Jul 25;6:30234. doi: 10.1038/srep30234 (PMC4958979; doi:10.1038/srep30234)
Supplement: Supplementary Information [file srep30234-s1.doc]

A simple Gateway-assisted construction system of TALEN genes for plant genome editing

Hiroaki Kusano, Hitomi Onodera, Miho Kihira, Hiromi Aoki, Hikaru Matsuzaki and Hiroaki Shimada

Department of Biological Science and Technology, Tokyo University of Science, Katsushika, Tokyo 125-8585, Japan

Supplemental Methods:

Construction of pPlat plasmids.

pPlat plasmids containing the Chloramphenicol resistant gene were constructed as follows. Using pDONR207 (Invitrogen, Carlsbad, USA), the fragments corresponding to three portions of the Chloramphenicol resistant gene were separately amplified using following primer sets: CAT-F_attB2 and CAT-B_Esp3I, CAT-A_BsaI and CAT-T_MscI, and CAT-S_MscI and CAT-R_AdTer. These fragments were connected to make a mutant Chloramphenicol gene at the Esp3I sites. The resultant Chloramphenicol resistant gene was amplified using primers, CAT-F_attB2 and CAT-R_AdTer. In parallel, the NOS terminator region was prepared from pCAMBIA1301 (CAMBIA, Australia) by PCR using primers, Ter-bhxF_attB1 and Ter-R_AdCAT. They were connected together and then amplified using primers CAT-F_attB2 and Ter-bhxF_attB1, by which the regions of attB1 and attB2 were introduced. Subsequently, the resultant fragment was inserted into pDONR207 by Gateway BP clonase to generate attL1 and attL2 sites. The attL1-attL2 region was amplified from the resultant plasmid using primers pPlatII-A_SpeI and pPlatII-I_SacI. This fragment was digested with SpeI and SacI, and ligated with the fragment containing the replication ori without *Esp*3I sites, which was prepared from pDONR207 by PCR using primers pPlatII-J_SacI and pPlatII-D_XbaI.

The platinum TALEN genes containing HD, NG, NI, and NN as RVD were constructed as follows: Each of platinum TALEN genes was prepared from ptCMV-136/63-VR-HD, NG, NI and NN plasmids, in which the TALEN gene was divided into N-terminal and C-terminal portions excluding 34-aa repeats at two *Esp*3I recognition sites, by PCR using primers TALEN63-F_BglII and TALEN63-R_HindIII. They were inserted between the BglII and HindIII sites of above-mentioned plasmids. PCR primers used in this study are shown in Supplementary Table 1.

pPlat plasmids containing the Ampicillin resistant gene were constructed by the similar way, using pGEX-3X (GE Healthcare Japan, Tokyo, Japan), and PCR primers; Amp-F_attB2, Amp-R_AdTer, Ter-R_AdAmp, pPlatII-C_EcoRI and pPlatII-B_EcoRI instead of pDONR207, CAT-F_attB2, CAT-R_AdTer, Ter-R_AdCAT, pPlatII-I_SacI and pPlatII-J_SacI, respectively.

Construction of pDual35SGw1301

Two fragments containing Gateway acceptor units were amplified from pGWB2 using primer sets, 35S-C_KpnI and attR2-R_SacI, and attR1-F_KpnI and attR2-R_HindIII, and they were inserted between the SacI and HindIII sites of pCAMBIA1301 (acc. AF234297) to generate a plasmid containing two sets of attR sites (pCAM-attR). A CaMV 35S promoter region together with the 3' portion of LacZα gene and the 5' portion of LacZα gene were prepared by PCR from pCAMBIA1301 using primer sets, LacZ-S and 35S-Z_XbaI, and LacZ-T and LacZ-A_EcoRI. They were fused by PCR using primers LacZ-A_EcoRI and 35S-Z_XbaI. The other CaMV 35S promoter was prepared from pCAMBIA1301 by PCR using primers 35S-D_EcoRI and 35S-Z_KpnI. These fragments were inserted between KpnI and XbaI sites of the pCAM-attR (pDual35SGW1301). The fragment including CaMV 35S promoter regions and Gateway acceptor unit was prepared by digestion with SacI and HindIII in pDual35SGw1301, and then introduced into pCAMBIA1300 to construct pDual35SGW1300. PCR primers used in this study are shown in Supplementary Table 1.

Construction of pSSARL-GwBP

The plasmid for pSSA assays containing a divided luciferase gene (divided Luc), in which a target sequence was inserted, were constructed as follows: A fragment of 35S promoter was amplified from pCAMBIA1301 by PCR using a primer set, 35S-A_PstI and 35S-B_SacI. A fragment encoding the Renilla luciferase, used as an internal standard in the SSA assay, was amplified from a plasmid of R-TK plasmid in Pica Gene Dual Sea Pansy Luminescence kit (Toyo-B-net, Japan) by PCR using a primer set, RLuc-F_SacI and RLuc-R_ApaI. A fragment for the divided Luc gene and the Nos terminator was chemically synthesized. This region contained the fragment corresponding to the 1st to 1035th nucleotide position of the ORF and the fragment for the 605th position and later of the ORF in the firefly luciferase gene (Luc), which contained a direct repeat of a 430-bp in the middle of the Luc gene. DNA synthesis was performed by standard service of Eurofin genomics (Eurofin Japan, Tokyo, Japan). This was amplified by PCR using a primer set, 2ALuc-F_ApaI, and NOS-R_KpnI. The PCR primer 2ALuc-F_ApaI contained the sequence for the 2A peptide. The resultant fragments were digested by the appropriate restriction enzymes, ligated together, and then inserted between the PstI and KpnI sites in pBluescriptII SK+. The Gateway attP1/2 cloning unit was amplified by PCR using primers attP1-BglII and attP2-XhoI from pDONR-207 (Invitrogen) as a template, and then inserted into the BglII and XhoI sites located at the border of two parts of the divided Luc gene. The resultant plasmid was named pSSARL-GwBP. The nucleotide sequence of the pSSARL-GwBP is shown in Supplementary Figure. The target sequences (50-nucleotides) that were expected to be recognized by the desired TALENs were chemically synthesized. These fragments were subjected to PCR amplification using the primer sets, StGBSS1-F_attB1 and StGBSS1-R_attB2, and StGBSS3-F_attB1 and StGBSS3-R_attB2, respectively. The resultant fragments were introduced into the pSSARL-GwBP using BP clonase. PCR primers used in this study are shown in Supplementary Table 1.

Plant transformation and preparation of the fragment containing the target region in the potato GBSS gene

Transformation of potato cells was performed by the Agrobacterium-mediated transformation method. *Agrobacterium tumefaciens* EHA105 strain was used for the host for TALEN genes in the destination vector, and infected the stem sections of potato, cultivar "Sayaka". Potato sections were cultured and induced callus on the following media: MS salts, 3% sucrose, 0.2% Gelrile, 1 mg/L Thiamine-HCl, 0.5 mg/L Nicotinic acid, 0.5 mg/L Pyridoxin-HCl, supplemented with 5 mg/L Hygromycin, 1.75 mg/L zeatin-riboside, 0.53 mg/L indol-acetic acid. More than one hundred Hygromycin-resistant potato callus were obtained from independent sections. The fragment corresponding to the region containing the target sequence in the *GBSS* gene was amplified by PCR using primers, 5'-CCCCTCGAGCTTGCCTACTGTAATCGGTGATAA-3' and 5'-CCCGGATCCCAAGCTGAACCTAAGTTCAT-3'.

Supplementary Table 1. Primers used in this study

　Name Sequence

Construction of pDual series

35S-C KpnI 5'- CAAGGTACCGGTCCCCAGATTAGCCT -3'

attR2-R_SacI 5'- CCCGAGCTCCGCTGTTATCAACCACTT -3'

attR1-F_KpnI 5'- TTTAAGCTTCTGGATGGCAAATAATGAT -3'

attR2-R_HindIII 5'- CCCAAGCTTCGCTGTTATCAACCACTT -3'

35S-D_EcoRI 5'- CCCGAATTCGCTTCATGGAGTCAAAGATT -3'

35S-Z_KpnI 5'- CAAGGTACCGGTCAAGAGTCCCCCGTGTT -3'

LacZ-S 5'- ATGACCATGATTACGAGCTTGGCACTGGC -3'

35S-Z_XbaI 5'- CAATCTAGAGGTCAAGAGTCCCCCGTGTT -3'

LacZ-T 5'- GCCAGTGCCAAGCTCGTAATCATGGTCAT -3'

LacZ-A_EcoRI 5'- CCCGAATTCACGACAGGTTTCCCGACTG -3'

Construction of pPlat series

CAT-F_attB2 5'-GGGGACCACTTTGTACAAGAAAGCTGGGTC

GACGGAAGATCACTTCGCA -3'

CAT-B_Esp3I 5'- GGAACCTCTTACGTGCCGATCAA -3'

CAT-A_BsaI 5'-TTGGCGAAAATGAGACCTTGATCGGCACGT

AA-3'

CAT-T_MscI 5'- TATTGGCCACGTTTAAATCAAAACTGGTGAA

ACTCACCCAGGGATTGGCTGACACGAA -3'

CAT-S_MscI 5'- AACGTGGCCAATATGGACAACTT -3'

CAT-R_AdTer 5'- TTGACAGGATATATTGGCAGCCATCCCTTCC

TGAT -3'

Ter-bhxF_attB1 5'-GGGGACAAGTTTGTACAAAAAAGCAGGCTTC

AGATCTCTAAGCTTCGTCTAGATCGTTCAAACA

TTTGGCAA-3'

Ter-R_AdCAT 5'- AATCAGGAAGGGATGGCTGCCAATATATCCT

GTCAA -3'

Ter-R_AdAmp 5'-AAGGATCTAGGTGAAGCCAATATATCCTGTC

AA-3'

Amp-F_attB2 5'-GGGGACCACTTTGTACAAGAAAGCTGGGTC

GACCCAGTCACGTAGCGATA -3'

Amp-R_AdTer 5'-TTGACAGGATATATTGGCTTCACCTAGATCC

TT -3'

pPlatII-A_SpeI 5'- CCCACTAGTAGCATGGATCTCGG -3'

pPlatII-C_EcoRI 5'- CCCGAATTCAACGCGAGAGTAGGGAACT -3'

pPlatII-B_EcoRI 5'- CCCGAATTCTGGATGGCAAATAATGAT -3'

pPlatII-D_XbaI 5'- CCCTCTAGACTGGCCCGTGTCTCAA -3'

pPlatII-I_SacI 5'- CCCGAGCTCTGGATGGCAAATAATGAT -3'

pPlatII-J_SacI 5'- CACGGGCCAGAGCGAGCTCTGGATGGCA -3'

TALEN63-F_BglII 5'- CCCAGATCTATGGACTATAAGGACCACGA -3'

TALEN63-R_HindIII 5'- CCCAAGCTTTTATGAGCGGAAATTGATCT -3'

Construction of pSSARL-GwBP

35S-A_PstI 5'- CCGCTGCAGGCTTCATGGAGTCAAAGATT -3'

35S-B_SacI 5'- TTTGAGCTCAAGAGTCCCCCGTGTTCT -3'

RLuc-F_SacI 5'-CTTGAGCTCAGAATGACTTCGAAAGTTTATG-3'

RLuc-R_ApaI 5'- GCTGGGCCCCTTGTTCATTTTTGAGAACT -3'

2ALuc-F_ApaI 5'- ACAAGGGGCCCAGCTGTTGAATTTTGACCTT

CTTAAACTAGCGGGAGATGTCGAGTCCAACC

CTGGACCTGAATTCGCCACCATGGAAGACG

CCAA-3'

NOS-R_KpnI 5'-AAAGGTACCTCTAGAATATCCTGTCAAACAC

TGAT -3'

attP1-BglII 5'-GGGAGATCTGAGACGTTGGGCCCCAAATAA

TGAT -3'

attP2-XhoI 5'- GGGCTCGAGGAGACGAAGCTTCTGGATGGC

AAATAATGAT -3'

StGBSS1-F_attB1 5'-GGGGACAAGTTTGTACAAAAAAGCAGGCTC

CCGTCTCGGATCCTCACAATGGTTTAAG -3'

StGBSS1-R_attB2 5'- GGGGACCACTTTGTACAAGAAAGCTGGGTCG

CGTCTCGTCGACTTAGTTCTTGATTGG -3'

StGBSS3-F_attB1 5'-GGGGACAAGTTTGTACAAAAAAGCAGGCTC

CCGTCTCGGATCCTGTGATCCGCTACTT -3'

StGBSS3-R_attB2 5'-GGGGACCACTTTGTACAAGAAAGCTGGGTCG

CGTCTCGTCGACTTTCAATGCTGTCTC -3'

General Protocol for vector construction by Emerald–Gateway TALEN system.

Materials:

An appropriate pPlatA plasmid,

An appropriate pPlatC plasmid,

A destination vector plasmid pDual35SGw1301,

Appropriate plasmids for 34 amino-acids modules and intermediate arrays in Platinum Gate TALEN kit (addgene Kit #1000000043).

Reagents:

Restriction enzymes *Apa*I and *Xho*I,

*Bsa*I-HF (New England Biolabs, Inc.) and its 10x CutSmart buffer,

*Esp*3I (Thermo Fisher Scientific) and its 10x Tango buffer,

Quick Ligase (New England Biolabs, Inc.) and 10x T4 DNA ligase buffer,

Gateway LR clonase II (Invitrogen).

Day 1:

1. Prepare the Platinum Gate Step I reaction mixture according to the procedure of Platinum Gate TALEN kit.
2. Add restriction enzyme *Apa*I. (optional)
3. Incubate them in a thermal cycler, with ten times of a repeated cycle with 37˚C for 5 minutes and 16˚C for 10 minutes. (Step I reactions)
4. Digest with *Bsa*I at 50˚C for 60 minutes after addition of *Bsa*I-HF with an appropriate volume of 10x CutSmart buffer.
5. Heat treatment at 80˚C for 5 minutes, and then stand the mixture at 4˚C. (Step I products)
6. Transform *E. coli* cells using the Step I products according to the general procedure, spread them on LB agar plates supplemented with appropriate concentration of Spectinomycin and X-Gal, and incubate the plates at 37˚C overnight.

Addition of *Apa*I to the reaction mixture is optional, but this process may reduce the amount of unexpected products and increase the yield.

Day 2:

1. Pick the white colonies generated on the plates, and put them into the LB liquid medium supplemented with an appropriate concentration of Spectinomycin.
2. Culture them overnight at 37˚C with shaking.

Day 3:

1. Extract the plasmid DNA from the *E. coli* cells in a culture medium, and dissolved in water upto the concentration of 50 ng/µL.
2. Prepare the Platinum Gate Step II reaction mixture without ptCMV series vector plasmids in Platinum Gate kit, and combine with an appropriate pPlat plasmid, either pPlatA or pPlatC,.
3. Incubate them in a thermal cycler, with six times of a repeated cycle with 37˚C for 5 minutes and 16˚C for 10 minutes. (Step II reaction)
4. Digest with *Xho*I and *Esp*3I at 37˚C for 60 minutes after addition of an appropriate volume of 10x Tango buffer. (optional)
5. Incubation at 80˚C for 5 minutes, and then put the mixture at 4˚C. (Step II products)
6. Transform *E. coli* cells using the Step II products, spread them on a LB agar plate supplemented with appropriate concentrations of Ampicillin and Chloramphenicol, and X-Gal, and incubate the plates overnight at 37˚C.

An appropriate pPlat plasmid will be chosen depending on the nucleotide sequence of the target by the desired TALENs. *Xho*I digestion is optional, but this process may reduce the amount of unexpected products and increase the yield of the final products. Step II products may be evaluated by *Bam*HI and *Pst*I digestions, which may detect the desired fragments introduced.

Day 4:

1. Pick the white colonies generated on the plate, and put them into the LB liquid medium supplemented with an appropriate antibiotics, then culture them overnight at 37˚C wish shaking.

Day 5:

1. Extract the plasmid DNA from the *E. coli* cells in a culture medium, and dissolved in water upto the concentration of 50 ng/µL.
2. Prepare the Gateway LR reaction mixture containing LR clonase II, and combine it with the resultant plasmid by the Step II reaction along with pDual35SGw1301.
3. Incubate it at 25˚C for 60 minutes, to accomplish the recombination by LR clonase II.
4. Transform *E. coli* cells using the reaction mixture, spread them on a LB agar plate supplemented with appropriate concentrations of Kanamycin, Ampicillin, and Chloramphenicol, and X-Gal, and incubate the plate overnight at 37˚C.

Day 6:

1. Pick the blue colonies generated on the plate, put them into 2 x YT liquid media supplemented with an appropriate concentration of Kanamycin, and culture them overnight at 37˚C with shaking.

Day 7:

1. Extract the resultant plasmid DNA from the *E. coli* cell in the overnight culture medium, and purify it according to the general procedure.

Supplemental Figure:


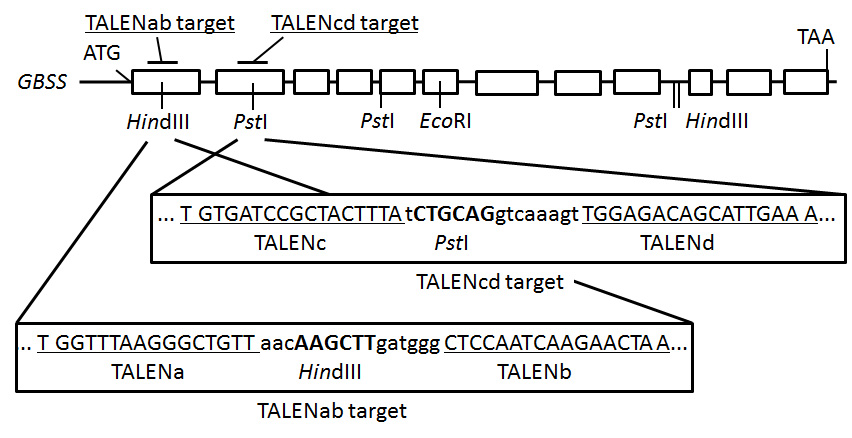


Supplemental Figure 1. Schematic representation of the target sites on the potato *GBSS* gene by the TALENs used in this study.

The responsible target sites of the TALENs are schematically represented on a map of the potato *GBSS* gene (NW_006238976: 91503-95114 region). Open boxes indicate exons. ATG and TAA indicate the start codon and stop codon, respectively, in the gene. Sites of representative restriction enzymes are indicated. “TALENab target” and “TALENcd target” show the positions of the target sites of the TALENs used in this work. Nucleotide sequences corresponding to these regions are shown below. The recognition sequences of TALENs are underlined with the names of TALENs, TALENa and TALENb, and TALENc and TALENd, respectively. Bold letters indicate the sites of the restriction enzymes.
